# Photon Up-Conversion Process to Test Media Ordering

**DOI:** 10.1021/acs.jpclett.6c00506

**Published:** 2026-04-28

**Authors:** Giulia Quaglia, Elena Cambiotti, Emiliano Fratini, Loredana Latterini

**Affiliations:** † Nano4Light Lab, Dipartimento di Chimica, Biologia e Biotecnologie, Università di Perugia, Via Elce di Sotto, 8, 06123 Perugia, Italy; ‡ Department of Chemistry “Ugo Schiff”, Via della Lastruccia 3, 50019 Sesto Fiorentino, Italy; § Consorzio per lo Sviluppo dei Sistemi a Grande Interfase (CSGI), Via della Lastruccia 3, 50019 Sesto Fiorentino, Italy

## Abstract

In this work, triplet–triplet annihilation upconversion (TTA-UC) was employed as a sensitive tool to study the influence of media organization on the efficiency of bimolecular processes. For this purpose, an archetypal sensitizer/emitter pair of platinum octaethylporphyrin (PtOEP) and 9,10-diphenylanthracene (DPA) was probed in oleic (OA) and elaidic acid (EA), chosen as isomeric phase change materials (PCMs). TTA-UC was monitored below and above the melting points of the PCMs. The results point out that the arrangement of solid- and liquid-phase media imposes constraints able to assist TTA-UC, resulting in higher efficiency in the solid phase of OA. A green-to-blue UC quantum yield of up to 3.6% was observed under ambient conditions with incoherent and low power excitation. The role and the effects of media constraints were monitored through steady-state and time-resolved luminescence, small-angle X-ray scattering (SAXS), Raman and UV–vis spectroscopy and were rationalized in terms of PtOEP aggregation.

## Graphical abstract

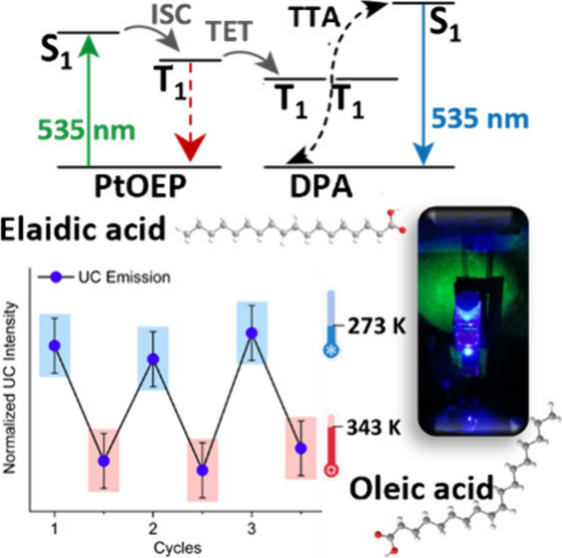

The development of devices requires optimized processes and processable materials. Often the optimization is carried out at a lab-scale, and the impact of media in conditioning the disposition and the molecular arrangement of active materials is not considered; therefore, the efficiency of the processes in the devices and their processability is underestimated. But the role of media in driving the molecular arrangements is particularly relevant when electronic excited states determine the performance of optoelectronic devices. However, extremely sensitive tools to monitor the role of media on the photophysical behavior of active molecules are necessary.

Strategies to convert low-energy radiation into higher-frequency light (i.e., upconversion, named UC) are receiving significant attention from the scientific community due to their potential to enhance radiation harvesting and exploitation.^1–6^ UC comes into the spotlight because of its wide technological applications that range from photocatalysis,^1,7–9^ solar cells,^10–12^ to biosensing, bioimaging,^13–15^ and photopharmacology.^16^ A versatile strategy to achieve UC emission is based on triplet–triplet annihilation (TTA) process.^17,18^ TTA-UC is a nonlinear optical process in which two dyes, with specific electronic properties, are involved.^19^ The sensitizer absorbs the low energy radiation and undergoes an efficient intersystem crossing to populate its triplet state; then, the absorbed energy is transferred through triplet–triplet energy transfer (TTET) to the triplet excited state of the emitter unit. This step generally occurs through short-range interactions (Dexter energy transfer), since it requires the overlap of sensitizer and emitter wave functions within 10 Å.^18,19^ Then, the collision between two emitter molecules in their triplet state results in annihilation; consequently, one emitter molecule returns to the ground state, while the other is promoted to the singlet excited state resulting in UC emission even under incoherent excitation.^2^ The TTA-UC process strongly relies on bimolecular processes between electronic excited states with properly selected energies and decay times.^20^ Dyes are frequently dissolved in liquid media, which ensure the dynamics to assist bimolecular interactions; however, not all solvents are adequate for the mechanism.^21,22^

Since TTA-UC requires an efficient energy transfer between sensitizer and emitter, it is a sensitive tool to investigate how the medium structuring affects the photophysical properties of dyes, which can be detrimental or assist the efficiency of the nonlinear process.^22^ In this regard, we focus our attention on oleic acid (OA) and elaidic acid (EA) used as media to disperse the chromophore pair. OA, a natural lipid, constitutes approximately 75% of the fat content in olive oil, while EA is commonly found in hydrogenated vegetable oil. Despite being used as sustainable media, OA and EA are geometrical isomers (C_18_H_34_O_2_, Figure 1); thus, we expected the same molecular interactions to occur with solutes. Their melting points close to room temperature (289 K for OA and 320 K for EA) classify them as organic nonparaffin phase change materials (PCMs) which can be switched between the liquid and solid state by small temperature changes. The employment of OA and EA to investigate the TTA-UC behavior of platinum octaethylporphyrin (PtOEP) and 9,10-diphenylanthracene (DPA) pair does not require deoxygenation treatment to avoid oxygen quenching of the triplet excited species thanks to their unsaturated bond, thus OA and EA are also interesting as promising oxygen scavenger.^23,24^ We examined whether the media impose constraints based on temperature variations and how their internal structure regulates the arrangement, disposition, and potential aggregation of chromophores, influencing the quantum efficiency of the overall photophysical process.

**Figure 1:**
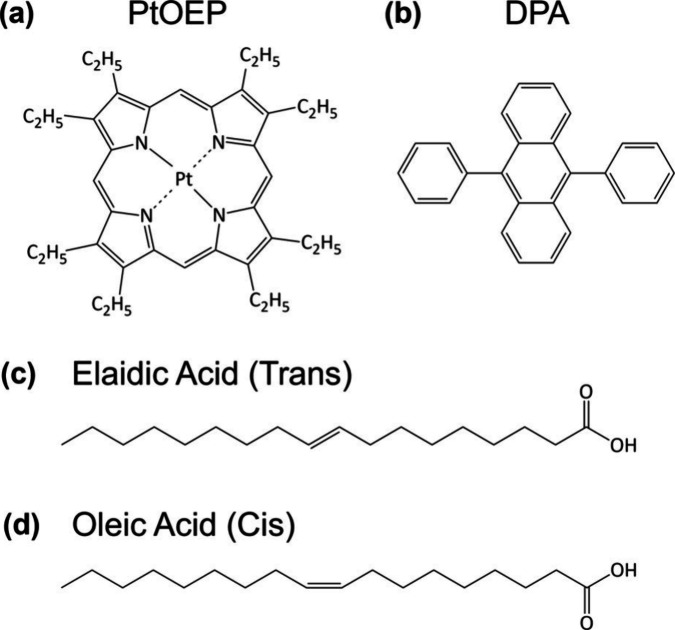
Chemical structure of: (a) PtOEP, (b) DPA, (c) elaidic, and (d) oleic acid.

A series of samples with varying PtOEP:DPA ratios were prepared, and the corresponding UC trends are listed in Figure 2. The PtOEP concentration was fixed at 1 mM, while DPA concentration was systematically varied. The results indicate that a ratio of 1:50 (1 mM of PtOEP and 50 mM of DPA) provides the highest UC emission in both solvents; therefore, this composition was selected for subsequent measurements.

**Figure 2:**
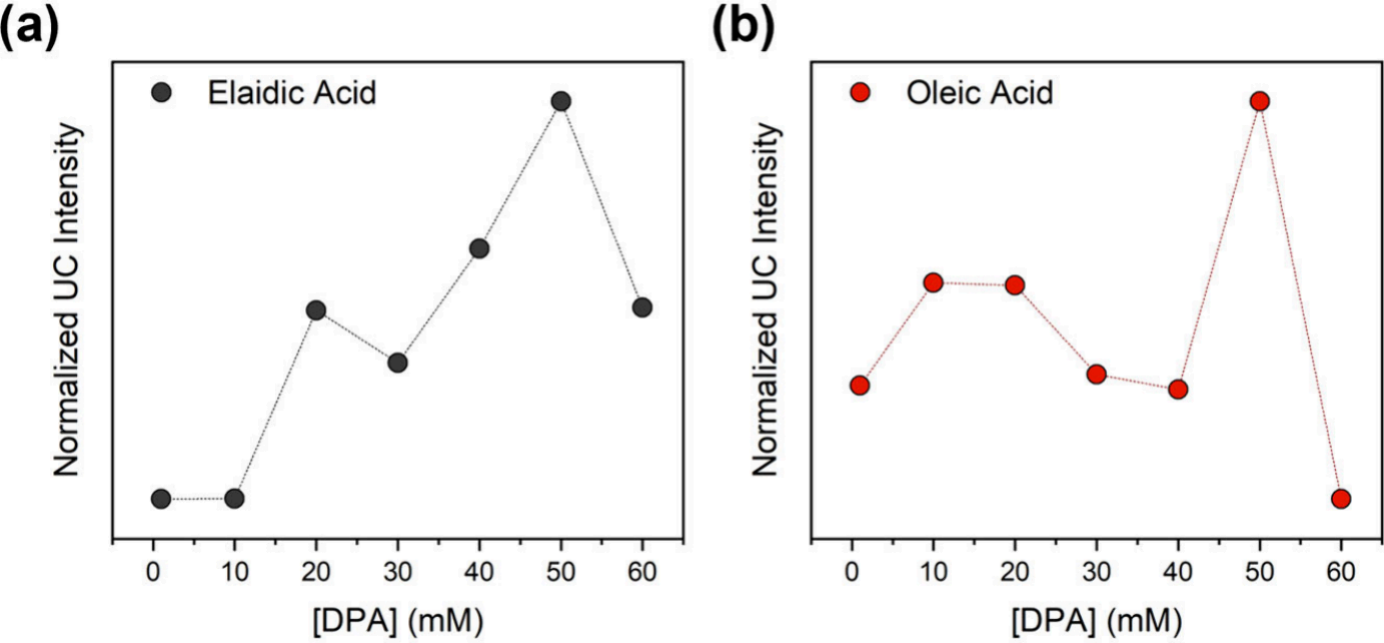
Upconversion emission in EA (a) and OA (b) at room temperature as a function of the DPA concentration with [PtOEP] fixed at 1 mM.

The TTA-UC emission was collected both above and below the melting point of the respective medium without previous deoxygenation. Given the similar chemical structure of the two media, we expected comparable molecular interactions with dyes and similar optical and photophysical behavior for the UC couple. However, as shown in Figure 3a-b, OA exhibits greater UC emission compared to EA. Table 1 summarizes the UC quantum yield (UCQY, determined by a relative method with inner-filter corrections; see the Supporting Information) for the respective samples in solid and liquid phase. A maximum UCQY value of 3.6% was measured in solid OA, which is relevant for TTA-UC in atmospheric conditions through an incoherent excitation source.^24^ These data reveal higher UCQY in the solid phase for both media despite restricted molecular diffusion. The reduced UC at high temperatures is related to the increased nonradiative deactivation of both the singlet and triplet excited states of DPA, as well as the conversion of generated DPA singlet states into intermolecular species, as reported by Goudzarti et al.^25^

**Table 1:** UC Quantum Yield in OA and EA at Solid and Liquid Phases as a Function of Chromophores Concentration

	UCQY OA		UCQY EA	
Sample	Solid	Liquid	Solid	Liquid
[PtOEP] = 1 mM/[DPA] = 50 mM	3.6%	2.4%	0.3%	0.1%
[PtOEP] = 0.12 mM/[DPA] = 6 mM	0.6%	0.2%	0.1%	0.05%

**Figure 3:**
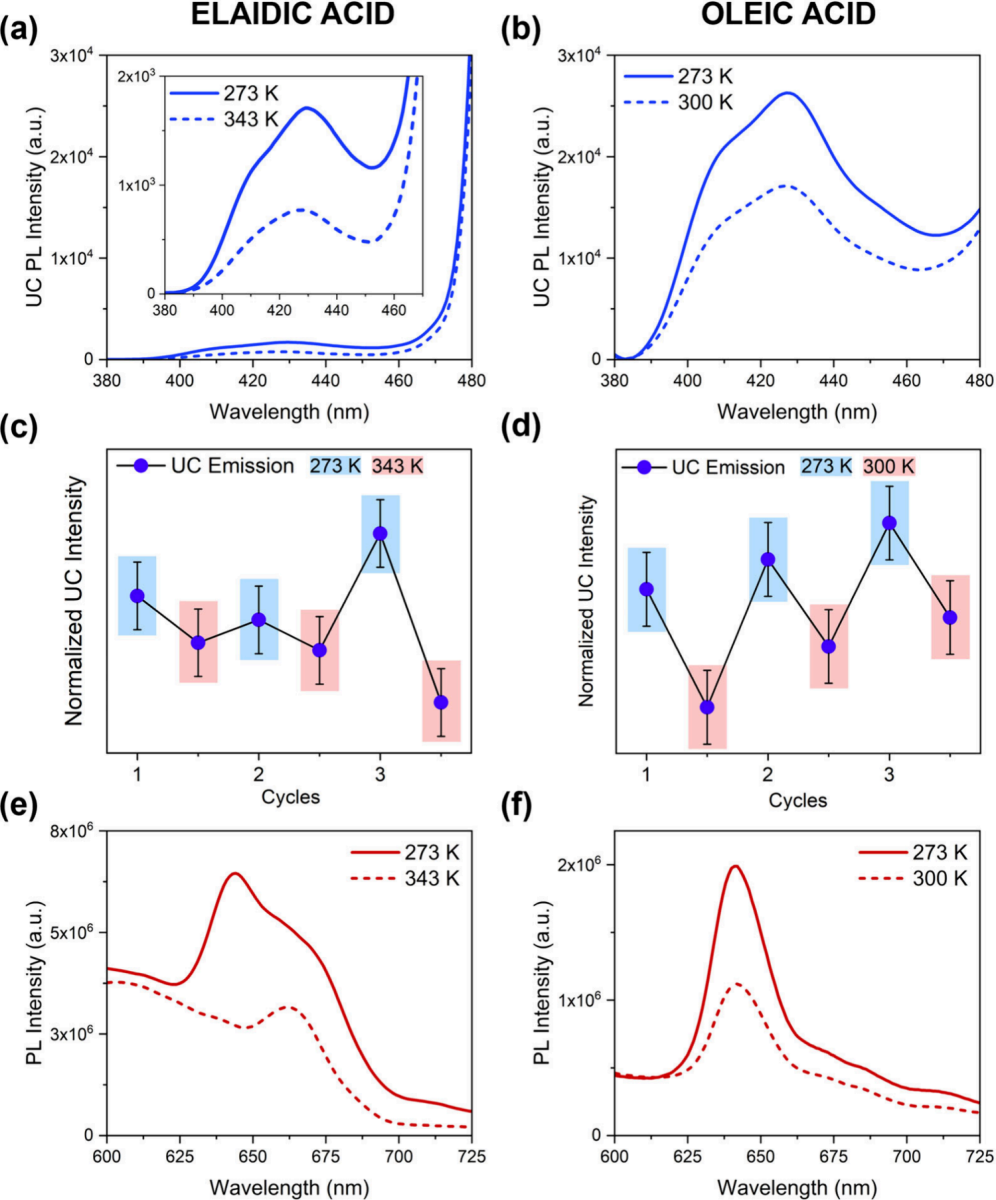
Upconversion emission (a,b) and phosphorescence spectra (e,f) in EA and OA with PtOEP in liquid (dashed line) and solid (solid line) phase. Schematic representation of UC intensity as a function of temperature in EA (c) and OA (d).

Furthermore, the UCQY in EA is about 20 times lower than in OA both considering the solid and liquid phases. These data indicate a relevant impact of media/aggregation state on the interchromophoric energy transfer processes,^26^ which results in a thermally regulated TTA-UC system. Control samples containing only DPA in OA and EA were prepared and measured under the same conditions used for the UC experiments. As shown in Figure S1, no DPA emission appeared, confirming that the observed emission originates exclusively from the UC process rather than from direct DPA excitation.

The effect of temperature was monitored with three solid–liquid cycles, during which UC emission was collected starting from low temperatures. Figure 3 panels c and d display a reproducible trend within the experimental error for both media.

To better rationalize the UC behavior in the two media, the phosphorescence spectrum of PtOEP in the UC dispersions was examined. In OA, the phosphorescence shows the profile of monomeric form of the sensitizer at 645 nm and it remains unchanged with temperature (Figure 3f).^27^ In EA, however, the spectrum presents an additional shoulder at longer wavelengths (660–680 nm), which is attributed to the formation of aggregates (Figure 3e). These species become predominant in the liquid phase, demonstrating that the formation of porphyrin aggregates is promoted by molecular diffusion.^28^ The UC emission was analyzed as a function of the excitation power density. From log–log plots, the incident power threshold (*I*_th_) was determined as the point of slope change, corresponding to the onset of TTA as the dominant triplet decay pathway. The extracted *I*_th_ values are 220 μW/cm^2^ for EA and 190 μW/cm^2^ for OA (Figure S2). The higher threshold in EA reflects the lower UC efficiency caused by PtOEP aggregation, whereas OA provides a more favorable environment for triplet migration and annihilation.

The absorption and reflectance spectra of PtOEP in the UC dispersions were acquired at room temperature in the 400–650 nm range, where excitation occurs (Figure S3a,b). In both media, S_0_–S_1_ PtOEP transition exhibits a structured band with maxima at 500 and 535 nm due to first vibronic Q(1) and pure electronic Q(0) transition, respectively.^27^ Moreover, a pronounced band at longer wavelengths (560 nm) can be observed in EA, which can be attributed to the S_0_–S_1_ transition in PtOEP aggregates, formed in the ground state.^27,29^ Upon decreasing the PtOEP concentration, the contribution of the 560 nm band is substantially reduced when EA is used as the medium, suggesting that the media isomerism imposes specific arrangements of the dye molecules able to alter their electronic transitions.

The shortening of PtOEP phosphorescence decay times (Figure S4) in EA, compared to the values measured in OA, further evidence the increase of nonradiative deactivation pathways likely due to aggregate formation.^30^ The TTET efficiencies estimated using $\Phi_{TTET}=1-\frac{\tau_{PtOEP/DPA}}{\tau_{PtOEP}}$ are comparable in the two media (94% in EA and 95% in OA).^31^ This small difference is insufficient to account for the pronounced variation in UCQY between OA and EA. Based on the above evaluation, it can be argued that the variations observed in UCQY between the two media may result from differences in PtOEP intersystem crossing (ISC) yield or the effectiveness of TTA for DPA in the two solvents.

To further investigate the role of aggregation, the phosphorescence of PtOEP is examined under identical UC conditions in the absence of DPA. In this case, PtOEP exhibits a markedly higher phosphorescence QY in OA (6.9%) than in EA (2.0%). The phosphorescence emission spectra are summarized in Figure S5 at different PtOEP concentrations. In EA, an additional shoulder centered at 675 nm is observed even at low PtOEP concentrations (0.012 mM) and becomes increasingly pronounced at higher PtOEP concentrations (Figure S5b). This is accompanied by an increased intensity ratio between the aggregates and the monomer peak. In contrast, OA exhibits a single sharp emission band at 645 nm, characteristic of monomeric PtOEP, with no appreciable spectral changes upon dilution (Figure S5a). Consistently, the normalized absorption spectra of PtOEP in the absence of DPA show no change in OA, while EA displays pronounced broadening of the PtOEP profile with increasing concentration (Figure S5c). This observation indicates that the aggregation does not arise from interactions with the annihilator.

To assess the impact of aggregation on UC performance, the study was extended to diluted dispersions while maintaining a constant PtOEP:DPA ratio (0.12 mM PtOEP, 6 mM DPA). The resulting UC spectra reproduce the behavior observed at higher concentration, with OA exhibiting greater efficiency in comparison to EA (Figure S6). As summarized in Table 1, dilution leads to an overall decrease in UCQY due to the reduced chromophore concentration. However, the OA/EA efficiency ratio decreases from ∼20 to ∼5, indicating that dilution suppresses aggregation in EA and yields a similar UC behavior in the two media. This trend is further supported by kinetic measurements (Figure S7), where diluted systems display longer decay times than the concentrated dispersions, consistent with reduced aggregation.

The influence of PtOEP aggregation on UCQY was evaluated using the absorbance difference (Δ*A*), which reflects the loss of monomeric PtOEP associated with aggregate formation. Δ*A* is defined as the difference between the measured absorbance and the expected absorbance for monomeric PtOEP, calculated using the experimentally determined molar extinction coefficients (ε = 15100 M^–1^ cm^–1^ in OA and ε = 11495 M^–1^ cm^–1^ in EA at 535 nm). As shown in Figure S8, an increase in Δ*A* for EA is systematically accompanied by a pronounced decrease in UCQY, indicating that a greater loss of monomeric PtOEP is associated with reduced upconversion efficiency. In contrast, OA maintains higher UCQY values under comparable conditions, consistent with lower aggregate formation.

To better understand the constraints of the cis/trans alkyl chain of the media, Raman spectra were collected on chromophores powders, pure PCMs, and the respective PCMs/chromophores dispersions. In both the OA and EA systems, the Raman spectra of the dispersions do not exhibit detectable signals of DPA and PtOEP (Figure 4). This is because, under the investigated conditions, the chromophore concentration is below the detection limit of the instrumental setup and/or their signals are masked by the fatty acid peaks. Interestingly, in the Raman spectra of EA (Figure 4a), significant changes are noted with the addition of DPA and PtOEP, while for OA they remain unchanged (Figure 4b). In EA, the intensity of the peaks at 1100 cm^–1^, assigned to the C–C symmetric stretching from the COOH and CH_3_ sides and at ∼1300 and ∼1450 cm^–1^, containing information regarding the conformational order of the hydrocarbon chains,^32,33^ are modified with the addition of chromophores, suggesting the formation of aggregates.

**Figure 4:**
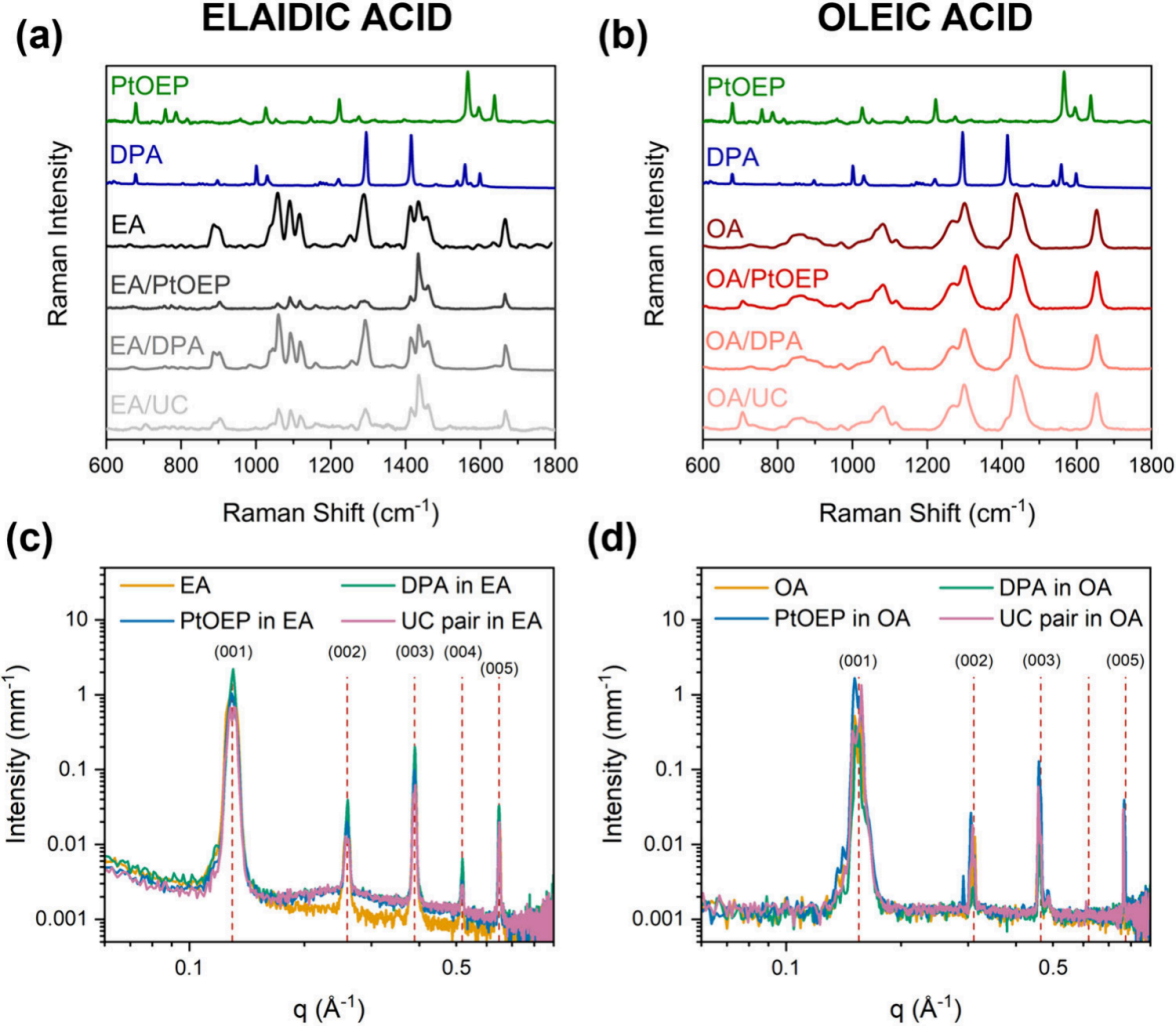
Raman spectra at environmental temperature (a,b) and SAXS patterns (c,d) at 276 K of pure PCMs, chromophore powders and dispersions composed by PCMs/chromophores for (a,c) elaidic acid and (b,d) oleic acid in solid phase.

The crystalline-phase arrangement of the two isomers was evaluated through small and wide-angle X-ray scattering (SWAXS) on the pure PCMs, PCMs with DPA, PCMs with PtOEP and PCMs containing the UC mixture of the two chromophores in both the solid and liquid state. Above the melting temperature of the respective media all the samples show the typical scattering associated to the structure factor of the fatty acids in the liquid state (Figure S9).^34^ The presence of the chromophores does not affect the SWAXS profile. Decreasing the temperature at 276 K, when the PCMs are in their solid state, the SAXS intensity distribution reveals at least five order reflections corresponding to the typical lamellar arrangement of fatty acids (Figure 4c,d). The lamellar lattice is characterized by a cell parameter (*d*-spacing) of 4.1 ± 0.1 nm for OA and 4.9 ± 0.1 nm for EA.^34,35^ These *d*-spacing values reflect variations in the chain length between the two isomers resulting from the cis/trans geometry.^36–38^ The EA-based system displays a marked increase in scattering intensity around 0.26 Å^–1^ upon addition of the chromophore addition. Subtraction of the neat EA contribution from the SAXS profiles of the chromophore-containing samples (Figure S10) reveals a broad scattering feature, consistent with the diffuse scattering typically associated with amorphous or partially fluid chain segments.^34^ These observations suggest a partial loss of the crystalline order and the presence of an amorphous liquid-like matrix, which can be induced by chromophore aggregation. In contrast, the presence of chromophores does not significantly alter the SAXS profiles of OA. The SAXS patterns as a function of temperature exhibit the solid–liquid transition of EA and OA (Figure S11), ensuring that the solvents were fully in the intended phase under experimental conditions.

In conclusion, in this work we investigate the effects of the media organization on the arrangement of solute molecules by monitoring the efficiency of complex photophysical processes, on which TTA-UC is based. Two isomeric hydrocarbons-based PCMs, oleic or elaidic acid, are employed as media for the study of TTA-UC between the PtOEP and DPA pair. The remarkable differences in UC quantum yield measured in OA and EA, either in liquid or solid phase (about 20-times higher in OA than EA) prompt a detailed characterization of the samples. Absorption and phosphorescence measurements evidence that PtOEP forms aggregates in EA, while no aggregate signals are detected for the OA sample. Given that the TTET efficiency exhibits consistent values (considering experimental error) in both solvents, the discrepancies measured in UCQY between the two solvents might stem from differences in the ISC quantum yields of PtOEP, influenced by aggregation; however, a decrease in the TTA in EA cannot be entirely dismissed at this point.

The modifications detected for EA in Raman and SAXS measurements, in the presence of PtOEP and DPA chromophores, indicated that the characteristic packing of the media impact the arrangement of the solute, affecting the interchromophoric interactions and hence the overall effectiveness of the UC process. The present data support an aggregation-associated suppression of upconversion in EA, while the precise relative contributions of ISC and TTA remain unresolved.

## Supplementary Material



## Notes

Detailed description of experimental synthesis, sample preparations, characterization methods, and UC quantum yield calculation. A table of refractive index values for the PCMs. Figures including: DPA emission as control sample, double logarithmic plot of UC as a function of incident power densities, reflectance and absorbance of UC samples in different media, phosphorescence and absorption spectra of PtOEP at different concentrations in absence of DPA, UC emission spectra in diluted condition, normalized phosphorescence decays of [PtOEP], correlation between absorbance difference and UCQY with the PtOEP concentration, SWAXS patterns of pure PCMs and in dispersion with chromophores, SAXS subtraction of the EA contribution, and the SAXS profiles of pure PCMs as a function of temperature (PDF)

## References

[1] Huang L., Wu W., Li Y., Huang K., Zeng L., Lin W., Han G. (2020). Highly Effective Near-Infrared Activating Triplet–Triplet
Annihilation Upconversion for Photoredox Catalysis. J. Am. Chem. Soc..

[2] Baluschev S., Miteva T., Yakutkin V., Nelles G., Yasuda A., Wegner G. (2006). Up-Conversion Fluorescence:
Noncoherent Excitation
by Sunlight. Phys. Rev. Lett..

[3] Zhu X., Su Q., Feng W., Li F. (2017). Anti-Stokes Shift Luminescent Materials
for Bio-Applications. Chem. Soc. Rev..

[4] Healy C., Hermanspahn L., Kruger P. E. (2021). Photon Upconversion in Self-Assembled
Materials. Coord. Chem. Rev..

[5] Richards B. S., Hudry D., Busko D., Turshatov A., Howard I. A. (2021). Photon Upconversion for Photovoltaics
and Photocatalysis:
A Critical Review: Focus Review. Chem. Rev..

[6] Bharmoria P., Bildirir H., Moth-Poulsen K. (2020). Triplet–Triplet Annihilation
Based near Infrared to Visible Molecular Photon Upconversion. Chem. Soc. Rev..

[7] Venkatesan P., Lin J.-Y., Roy D., Aloni P., Lin Z.-F., Doong R.-A. (2025). Enhanced Solar-Driven
Photoelectrocatalytic Water Treatment
and Hydrogen Evolution with Triplet-Triplet Annihilation Upconversion
with Mo-Doped BiVO4 Nanocomposite Films. Appl.
Catal. B Environ. Energy.

[8] Hwang S.-Y., Song D., Seo E.-J., Hollmann F., You Y., Park J.-B. (2022). Triplet–Triplet Annihilation-Based Photon-Upconversion
to Broaden the Wavelength Spectrum for Photobiocatalysis. Sci. Rep..

[9] Ravetz B. D., Pun A. B., Churchill E. M., Congreve D. N., Rovis T., Campos L. M. (2019). Photoredox Catalysis
Using Infrared Light via Triplet
Fusion Upconversion. Nature.

[10] Schulze T. F., Schmidt T. W. (2015). Photochemical Upconversion:
Present Status and Prospects
for Its Application to Solar Energy Conversion. Energy Environ. Sci..

[11] Dilbeck T., Hanson K. (2018). Molecular Photon Upconversion Solar Cells Using Multilayer
Assemblies: Progress and Prospects. J. Phys.
Chem. Lett..

[12] Carrod A. J., Gray V., Börjesson K. (2022). Recent Advances
in Triplet–Triplet
Annihilation Upconversion and Singlet Fission, towards Solar Energy
Applications. Energy Environ. Sci..

[13] Sun L.-D., Wang Y.-F., Yan C.-H. (2014). Paradigms
and Challenges for Bioapplication
of Rare Earth Upconversion Luminescent Nanoparticles: Small Size and
Tunable Emission/Excitation Spectra. Acc. Chem.
Res..

[14] Zhou J., Liu Q., Feng W., Sun Y., Li F. (2015). Upconversion Luminescent
Materials: Advances and Applications. Chem.
Rev..

[15] Liu Q., Xu M., Yang T., Tian B., Zhang X., Li F. (2018). Highly Photostable
Near-IR-Excitation Upconversion Nanocapsules Based on Triplet–Triplet
Annihilation for in Vivo Bioimaging Application. ACS Appl. Mater. Interfaces.

[16] Klimezak M., Chaud J., Brion A., Bolze F., Frisch B., Heurtault B., Kichler A., Specht A. (2024). Triplet-Triplet Annihilation
Upconversion-Based Photolysis: Applications in Photopharmacology. Adv. Healthc. Mater..

[17] Singh-Rachford T. N., Castellano F. N. (2010). Photon
Upconversion Based on Sensitized Triplet–Triplet
Annihilation. Coord. Chem. Rev..

[18] Cheng Y. Y., Fückel B., Khoury T., Clady R. G. C. R., Tayebjee M. J. Y., Ekins-Daukes N. J., Crossley M. J., Schmidt T. W. (2010). Kinetic
Analysis of Photochemical Upconversion by Triplet–Triplet Annihilation:
Beyond Any Spin Statistical Limit. J. Phys.
Chem. Lett..

[19] Gray, V. In Photon upconversion through triplet–triplet annihilation; Albini, A. , Protti, S. , Eds.; Photochemistry Vol. 47; Royal Society of Chemistry, 2019; pp 404–420, 10.1039/9781788016520-00404.

[20] Dzebo D., Moth-Poulsen K., Albinsson B. (2017). Robust Triplet–Triplet Annihilation
Photon Upconversion by Efficient Oxygen Scavenging. Photochem. Photobiol. Sci..

[21] Quaglia G., Campana F., Latterini L., Vaccaro L. (2022). Green Solvent Selection
for Green-to-Blue Upconversion Based on TTA. ACS Sustain. Chem. Eng..

[22] Collins A. R., Zhang B., Bennison M. J., Evans R. C. (2024). Ambient Solid-State
Triplet–Triplet Annihilation Upconversion in Ureasil Organic–Inorganic
Hybrid Hosts. J. Mater. Chem. C.

[23] Svagan A.
J., Busko D., Avlasevich Y., Glasser G., Baluschev S., Landfester K. (2014). Photon Energy Upconverting Nanopaper: A Bioinspired
Oxygen Protection Strategy. ACS Nano.

[24] Lee H., Lee M.-S., Uji M., Harada N., Park J.-M., Lee J., Seo S. E., Park C. S., Kim J., Park S. J., Bhang S. H., Yanai N., Kimizuka N., Kwon O. S., Kim J.-H. (2022). Nanoencapsulated
Phase-Change Materials: Versatile
and Air-Tolerant Platforms for Triplet–Triplet Annihilation
Upconversion. ACS Appl. Mater. Interfaces.

[25] Goudarzi H., Keivanidis P. E. (2014). Triplet–Triplet Annihilation-Induced Up-Converted
Delayed Luminescence in Solid-State Organic Composites: Monitoring
Low-Energy Photon Up-Conversion at Low Temperatures. J. Phys. Chem. C.

[26] Massaro G., Hernando J., Ruiz-Molina D., Roscini C., Latterini L. (2016). Thermally
Switchable Molecular Upconversion Emission. Chem. Mater..

[27] Bansal A.-K., Holzer W., Penzkofer A., Tsuboi T. (2006). Absorption and Emission
Spectroscopic Characterization of Platinum-Octaethyl-Porphyrin (PtOEP). Chem. Phys..

[28] Dienel T., Proehl H., Fritz T., Leo K. (2004). Novel Near-Infrared
Photoluminescence from Platinum (II)-Porphyrin (PtOEP) Aggregates. J. Lumin..

[29] Penconi M., Gentili P. L., Massaro G., Elisei F., Ortica F. (2013). A Triplet—Triplet
Annihilation Based up-Conversion Process Investigated in Homogeneous
Solutions and Oil-in-Water Microemulsions of a Surfactant. Photochem. Photobiol. Sci..

[30] Goudarzi H., Keivanidis P. E. (2017). All-Solution-Based
Aggregation Control in Solid-State
Photon Upconverting Organic Model Composites. ACS Appl. Mater. Interfaces.

[31] Raišys S., Adomėnienė O., Adomėnas P., Rudnick A., Köhler A., Kazlauskas K. (2021). Triplet Exciton
Diffusion and Quenching in Matrix-Free Solid Photon Upconversion Films. J. Phys. Chem. C.

[32] Kobayashi M., Kaneko F., Sato K., Suzuki M. (1986). Vibrational Spectroscopic
Study on Polymorphism and Order-Disorder Phase Transition in Oleic
Acid. J. Phys. Chem..

[33] Orendorff C. J., Ducey M. W., Pemberton J. E. (2002). Quantitative
Correlation of Raman
Spectral Indicators in Determining Conformational Order in Alkyl Chains. J. Phys. Chem. A.

[34] Tandon P., Förster G., Neubert R., Wartewig S. (2000). Phase Transitions in
Oleic Acid as Studied by X-Ray Diffraction and FT-Raman Spectroscopy. J. Mol. Struct..

[35] Ueno S., Suetake T., Yano J., Suzuki M., Sato K. (1994). Structure
and Polymorphic Transformations in Elaidic Acid (*Trans-ω*9-Octadecenoic Acid). Chem. Phys. Lipids.

[36] Wei Y., Li Z. (2016). Measurement
of D-Spacing of Crystalline Samples with SAXS. Measurement.

[37] Konieczkowska J., Siwy M. (2023). Comprehensive Investigations of Trans-Cis-Trans Isomerization in
the Solid State for Azo Polyimides. Dyes Pigments.

[38] Sievens-Figueroa L., Guymon C. A. (2009). Polymerization Kinetics
and Nanostructure Evolution
of Reactive Lyotropic Liquid Crystals with Different Reactive Group
Position. Macromolecules.

