# Supplementary material for: Photon Up-Conversion Process to Test Media Ordering
Source: J Phys Chem Lett. 2026 Apr 28;17(18):5288–93. doi: 10.1021/acs.jpclett.6c00506 (PMC13158986; doi:10.1021/acs.jpclett.6c00506)
Supplement: Supplementary file 1 [file jz6c00506_si_001.pdf]

Supporting Information for

Photon Up-Conversion Process to Test Media Ordering

Giulia Quaglia[a,‡], Elena Cambiotti[b,c,‡], Emiliano Fratini[b,c] and Loredana Latterini[a,c]*

[a]Nano4Light Lab, Dipartimento di Chimica, Biologia e Biotecnologie, Università di Perugia, Via Elce di Sotto, 8, 06123, Perugia, Italy

[b]Department of Chemistry "Ugo Schiff", Via della Lastruccia 3, 50019, Sesto Fiorentino, Italy

[c]Consorzio per lo Sviluppo dei Sistemi a Grande Interfase (CSGI), Via della Lastruccia 3, 50019, Sesto Fiorentino, Italy

‡These authors contributed equally to this work.

*Corresponding Author: **Loredana Latterini –** loredana.latterini@unipg.it

Contents Page

Experimental Section	3
Characterization Methods	3
Determination of the UC Quantum Yield	4
Experimental refractive index values of OA and EA	5.
Emission spectra of DPA in EA and OA collected under 532nm	5
Logarithmic plots of UC intensity versus incident power density for in EA and OA.	7
Absorption and reflectance spectra of UC dispersions in EA and OA	7
Phosphorescence decays of PtOEP in EA and OA	8
Normalized spectra of PtOEP dispersions in absence of DPA	9
Upconversion emission spectra in diluted solutions	10
Normalized phosphorescence decays of PtOEP in diluted solutions	10
Absorbance difference (ΔA) and UCQY versus PtOEP concentration	11
SWAXS patterns at 343K of pure PCMs and dispersions	11
SAXS raw spectra at 276K	12
SAXS profiles of EA and OA	12
Bibliography	13

Experimental Section

2,3,7,8,12,13,17,18-octaethyl-21H,23H-porphyrin platinum (II) (PtOEP), 9-10-diphenylanthracene (DPA), oleic acid (technical grade 90%), elaidic acid (technical grade ≥98%) and dichloromethane (CH_2Cl_2) were purchased from Sigma-Aldrich. All reagents and solvents were used as received without further treatment. The preparation of upconversion (UC) solutions was carried out starting from a stock solution of PtOEP and DPA in CH_2Cl_2 as solvent. Then, aliquots of stock solution were added to oleic (or elaidic) acid getting a final molar ratio of PtOEP/DPA=1:50 (with [PtOEP] equal to 1 mM or 0.12 mM). The UC solution was kept at 50°C overnight to completely remove all the CH_2Cl_2.PtOEP solutions in oleic (or elaidic) acid with final concentrations of 1 mM, 0.12 mM, 0.012 mM were prepared following the same procedure. All OA and EA samples were freshly prepared before measurements and handled with minimal exposure to air and light to reduce oxidation of the unsaturated fatty acids.

Characterization methods

Absorption spectra of the liquid samples were registered by a Cary 8454 UV–Vis Diode Array spectrophotometer (Varian) in a quartz cuvette with optical path of 1 cm. Reflectance spectra of the solid samples were recorded by a Cary 4000 spectrophotometer (Varian), equipped with a 150 nm integrating sphere (DRA-900). A barium sulphate tablet was used as reference. The photoluminescence spectra of the solutions were determined in a quartz cuvette with an optical path of 2 mm, using a Spex Fluorolog F112AI spectrophotometer (Horiba) equipped with a 450 W Xenon lamp. To record the upconversion emission, the excitation light at 535 nm passed through a 488 nm cut-off filter to avoid the direct excitation of the acceptor, while the emission radiation crossed a 532 nm notch filter to remove the incident radiation contribution. The emission spectra corrected for the instrumental response, were measured using a front-face geometry (22.5°). The UC emission spectra were corrected for the inner filter effects.[1] The excitation power-dependence of UC emission was investigated under the same measurement conditions as those used for UC spectra. The excitation intensity of the Xe lamp was controlled by introducing a series of neutral density filters into the excitation path. Temperature dependent emission experiments

were performed using an Oxford Instrument Cryostat. Emission decay profiles of PtOEP were acquired above the melting point using a pulsed diode source of the spectrofluorometer Edinburgh FS5 upon direct excitation at 510 nm. The Raman spectra at room temperature were acquired by a confocal Raman microscope (Olympus IX73 inverted microscope coupled to the S&I MonoVista CRS+spectrometer) exciting the sample at 785 nm through a 50×objective lens (numerical aperture, NA=0.50) and collecting the 500–3100 cm^{-1} spectral range with a resolution of 2 cm^{-1}. SAXS experiments were carried out through a Xeuss 3.0 HR apparatus (Xenocs, Grenoble), equipped with a high brightness X-ray tube, a FOX 3D single reflection multilayer optic, and a Dectris Eiger 2R 1M hybrid photon counting detector (pixel area = 75×75 μm^2). The X-ray beam corresponds to the Cu Kα radiation (λ = 1.542 Å) emitted by a micro-focus tube working at full power (30 W). The experiments were performed at two sample-to-detector distances, 290 and 1790 mm. More specifically, at first, 2D SAXS images were collected and 1D data, expressed as Intensity vs. the scattering vector, q (i.e. $q=(4\pi/\lambda)\ sin\theta$; 2θ is the scattering angle) were obtained by circularly averaging 2D images. Thus, data correction was performed by empty holder subtraction, followed by merging the scattering profiles obtained at the two sample-to-detector distances. All data reduction was performed trough XSACT software (Xenocs, Grenoble). All the samples are investigated in air equilibrated environment using a Peltier capillary holder where the temperature was set at 3°C. Silver behenate was used to calibrate the sample-detector distance according to standard procedures.[2] The intensity was then converted in absolute scale, using the scattering profile of a calibrated Glassy carbon acquired in the same experimental conditions and knowing the sample thickness.[3] Refractive index values of the two media were collected through a Schmidt + Haensch VAriRef refractometer with temperature control.

Determination of the UC Quantum Yield

The quantum yield (QY) is quantified following the indication given by IUPAC, that implies a maximum of 50%.[4] The UC QY is determined, through the following equation, using Rhodamine B in ethanol) as standard:

$$\Phi_{UC} = \Phi_F^{STD} \cdot \left(\frac{A_{STD}}{A_S}\right) \cdot \left(\frac{I_S}{I_{STD}}\right) \cdot \left(\frac{n_S}{n_{STD}}\right)^2$$

where Φ_F^{STD} is the emission QY of the standard (i.e. 0.7), A_{STD} and A_S are the absorbances at the excitation wavelength of the standard and the sample respectively, I_{STD} and I_S are the integrated emissions of the standard and of the sample while n_{STD} and n_S are their experimental refractive index values (Table S1). Since we are working with high sensitizer concentrations, the primary inner filter effect must be considered.[5] In these conditions, part of the emitted light is reabsorbed by the sample, resulting in an underestimation of the actual UC signal detected by the photomultiplier. To address this issue, several correction strategies have been developed. In particular, Lakowicz introduced a method to account for the re-absorption of both the incident and emitted radiation.[6] The corrected emission can be calculated using the following equation:

$$I_{Corr} = I_{Obs} \cdot antilog\,(\frac{A_{Exc} + A_{Emi}}{2})$$

where I_{Corr} is the corrected intensity, I_{Obs} is the observed UC emission intensity, A_{Exc} and A_{Emi} are the absorbance values at the excitation wavelength and the UC emission maximum, respectively.

Figures and Table

Table S1. Experimental refractive index values of OA and EA in the solid and liquid phase. The refractive index of solid OA exceeded the instrumental measurement limit.

Sample	OA		EA	
	**Solid**	**Liquid**	**Solid**	**Liquid**
Refractive index	N/A	1.45783	1.57479	1.44846

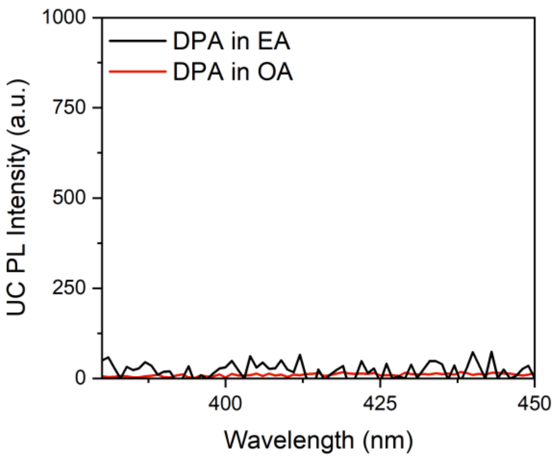

Figure S1. Emission spectra of DPA in EA (black line) and OA (red line) collected under 532 nm excitation with 488 nm long-pass filter.

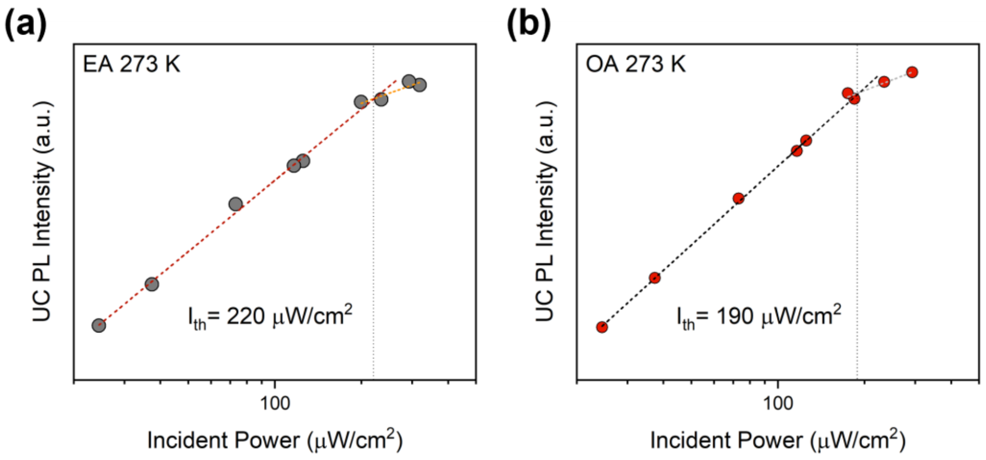

Figure S2. Double logarithmic plots of UC intensity versus incident power density for PtOEP/DPA in EA (a) and OA (b) at 273 K.

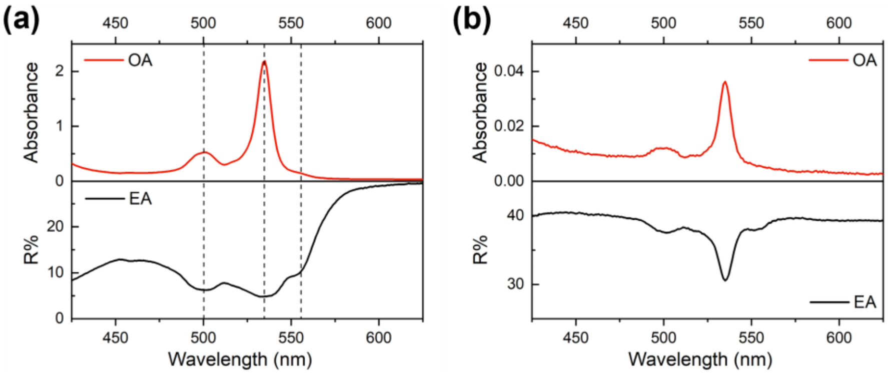

Figure S3. Absorbance and reflectance spectra of UC dispersions with [PtOEP]=1 mM (a) and [PtOEP]=0.012 mM (b) in elaidic (black line) and oleic (red line) acid.

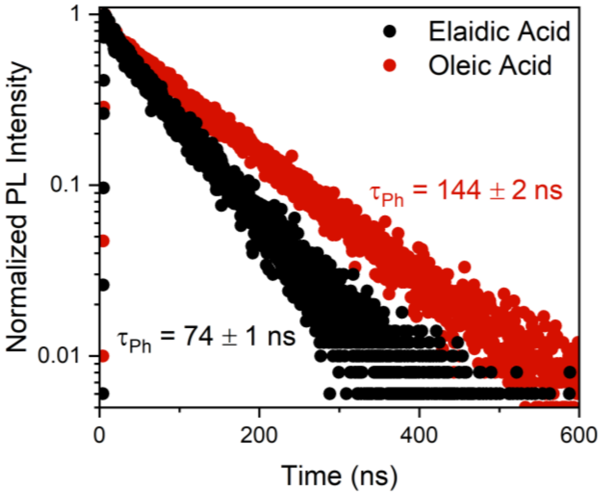

Figure S4. Normalized phosphorescence decays of [PtOEP]=1 mM in EA (black) and OA (red) in liquid phase registered at 645 nm upon excitation at 510 nm.

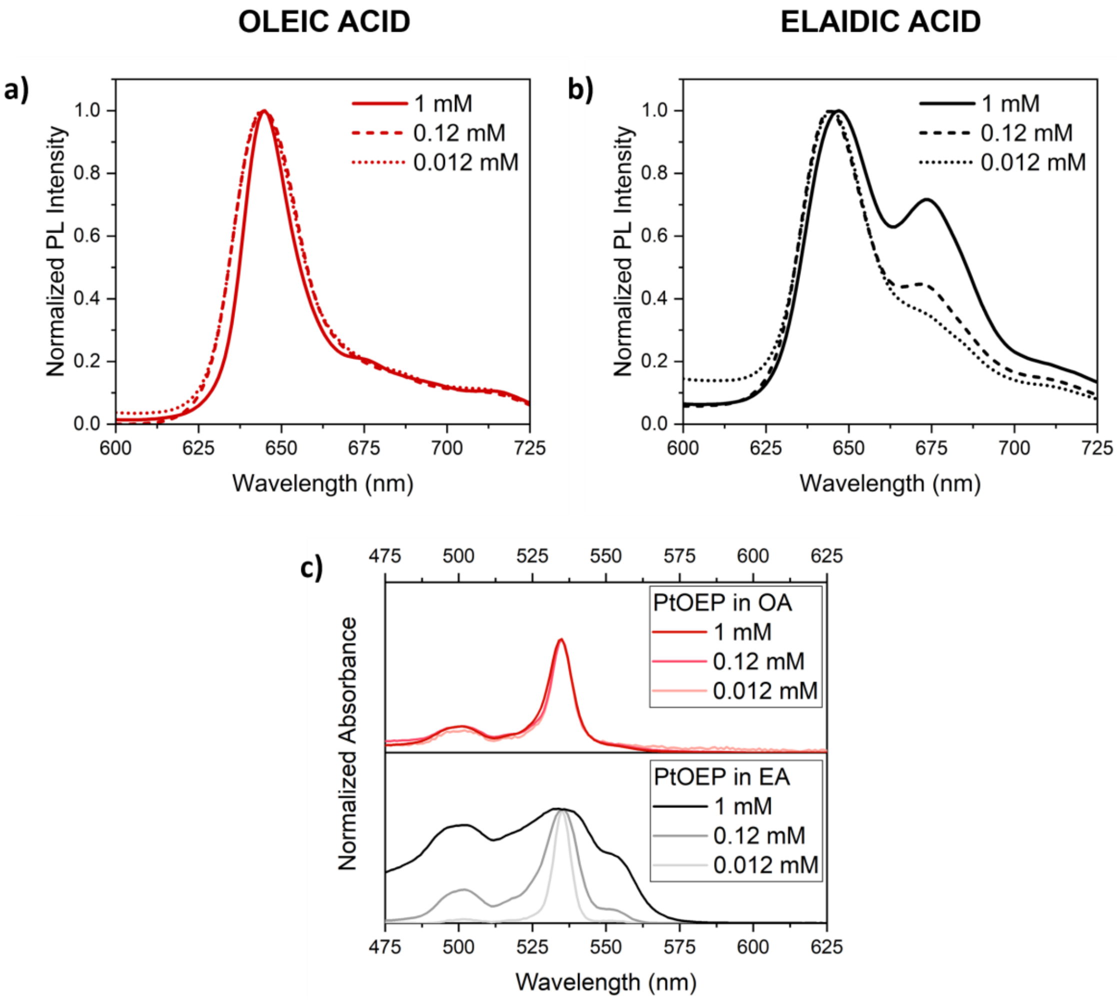

Figure S5. Normalized spectra of PtOEP dispersions in absence of DPA for different sensitizer concentrations (1, 0.12, and 0.012 mM) at room temperature. Phosphorescence spectra in (a) oleic acid and (b) elaidic acid, and (c) absorption spectra.

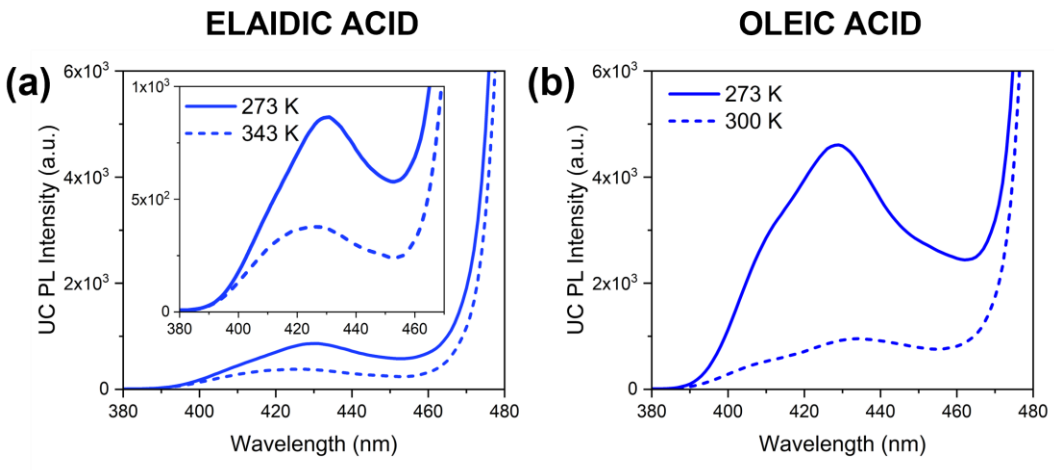

Figure S6. Upconversion emission spectra in diluted condition ([PtOEP]=0.12 mM) for elaidic (a) and oleic acid (b) in liquid (dash line) and solid (solid line) phase.

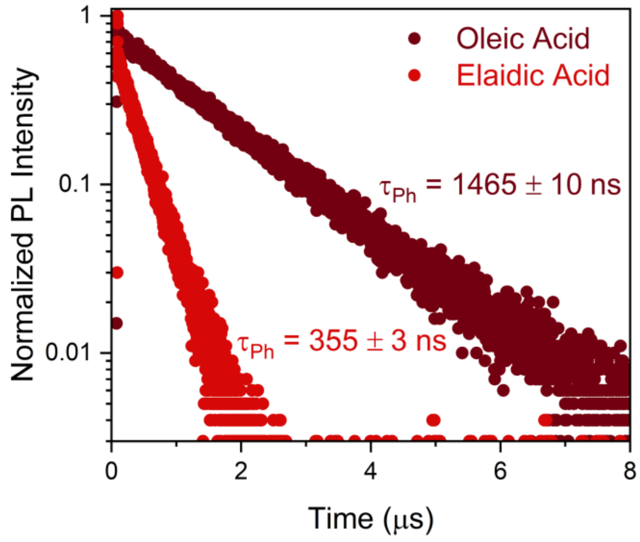

Figure S7. Normalized phosphorescence decays of [PtOEP]=0.12 mM in EA and OA in liquid phase registered at 645 nm upon excitation at 510 nm.

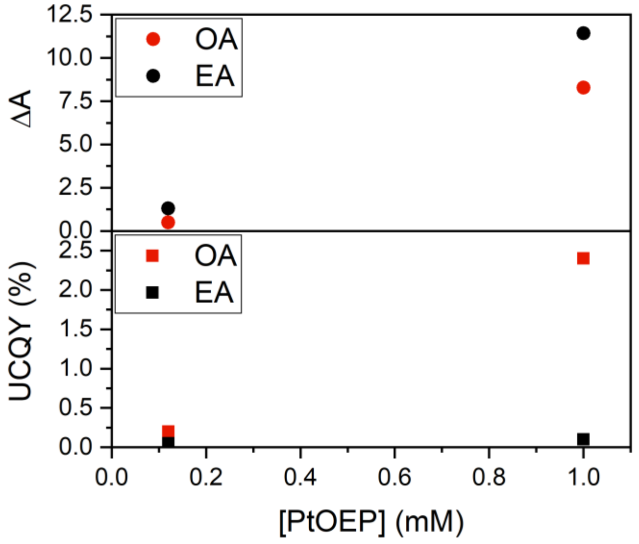

Figure S8. Absorbance difference (ΔA) and UCQY versus PtOEP concentration.

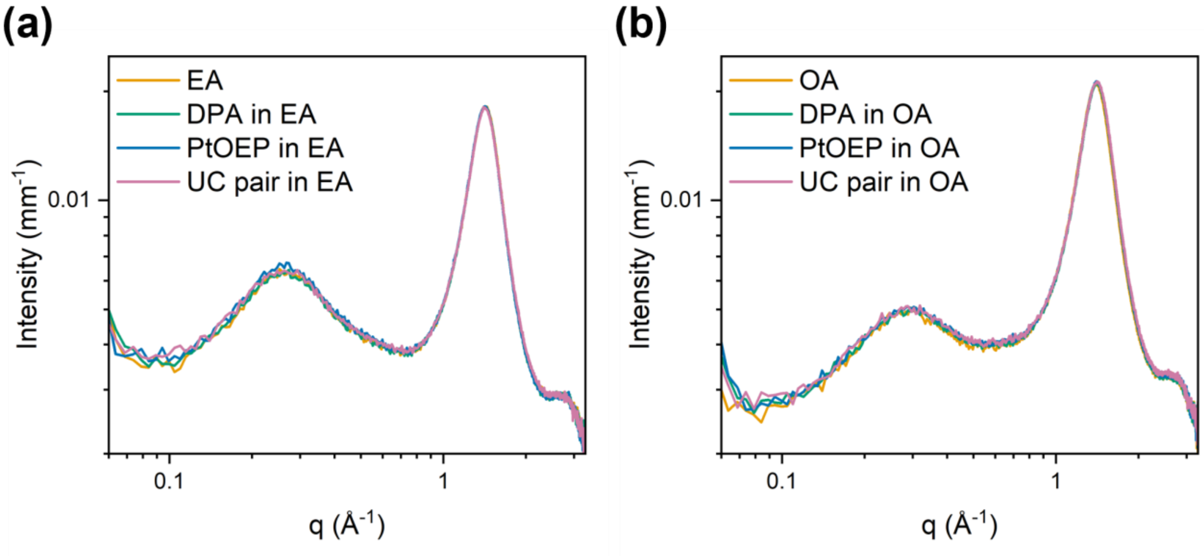

Figure S9: SWAXS patterns at 343K of pure PCMs and dispersions composed by PCMs/Chromophores for (a) elaidic acid and (b) oleic acid in liquid-state.

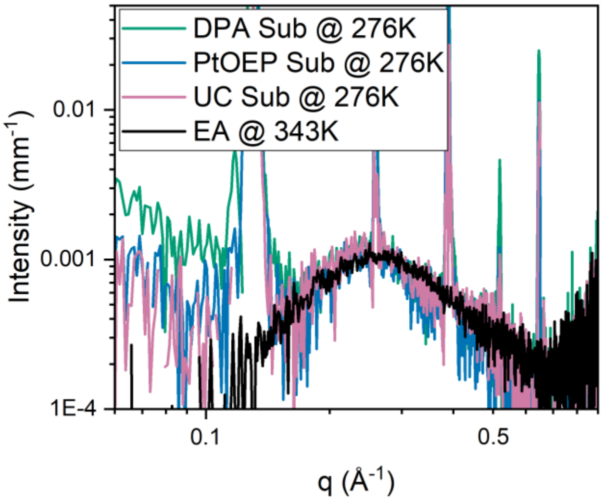

Figure S10. SAXS subtraction of the EA contribution at 276K from chromophore-containing samples and comparison with the amorphous EA at 343K (black pattern).

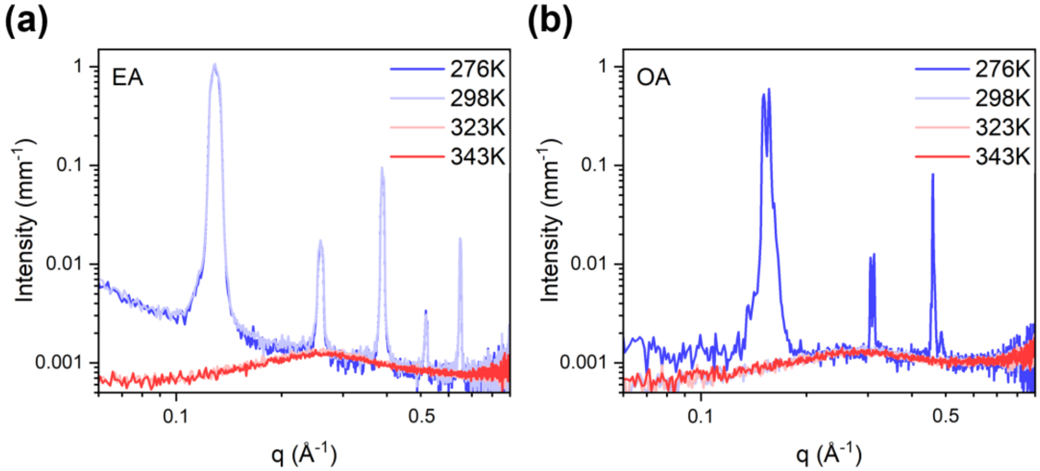

Figure S11. SAXS profiles of pure PCMs as a function of temperature: (a) elaidic and (b) oleic acid.

Bibliography

(1) Lakowicz, J. R. *Topics in Fluorescence Spectroscopy: Nonlinear and Two-Photon-Induced Fluorescence*; Springer Science & Business Media, 2006; Vol. 5.

(2) Blanton, T. N.; Huang, T. C.; Toraya, H.; Hubbard, C. R.; Robie, S. B.; Louer, D.; Göbel, H. E.; Will, G.; Gilles, R.; Raftery, T. JCPDS—International Centre for Diffraction Data Round Robin Study of Silver Behenate. A Possible Low-Angle X-Ray Diffraction Calibration Standard. *Powder Diffr.* **1995**, *10* (2), 91–95.

(3) Zhang, F.; Ilavsky, J.; Long, G. G.; Quintana, J. P. G.; Allen, A. J.; Jemian, P. R. Glassy Carbon as an Absolute Intensity Calibration Standard for Small-Angle Scattering. *Metall. Mater. Trans. A* **2010**, *41* (5), 1151–1158. https://doi.org/10.1007/s11661-009-9950-x.

(4) McNaught, A. D.; Wilkinson, A. *Compendium of Chemical Terminology*; Blackwell Science Oxford, 1997; Vol. 1669.

(5) Credi, A.; Prodi, L. From Observed to Corrected Luminescence Intensity of Solution Systems: An Easy-to-Apply Correction Method for Standard Spectrofluorimeters. *Spectrochim. Acta. A. Mol. Biomol. Spectrosc.* **1998**, *54* (1), 159–170. https://doi.org/10.1016/S1386-1425(97)00224-2.

(6) Instrumentation for Fluorescence Spectroscopy. In *Principles of Fluorescence Spectroscopy*; Lakowicz, J. R., Ed.; Springer US: Boston, MA, 2006; pp 27–61. https://doi.org/10.1007/978-0-387-46312-4_2.
